# Replication of Previous Genome-wide Association Studies of Bone Mineral Density in Premenopausal American Women

**DOI:** 10.1002/jbmr.62

**Published:** 2010-02-08

**Authors:** Shoji Ichikawa, Daniel L Koller, Leah R Padgett, Dongbing Lai, Siu L Hui, Munro Peacock, Tatiana Foroud, Michael J Econs

**Affiliations:** 1 Department of Medicine, Indiana University School of MedicineIndianapolis, IN, USA; 2 Department of Medical and Molecular Genetics, Indiana University School of MedicineIndianapolis, IN, USA

**Keywords:** bone mineral density, genome-wide association study, linkage disequilibrium, replication, single-nucleotide polymorphism

## Abstract

Bone mineral density (BMD) achieved during young adulthood (peak BMD) is one of the major determinants of osteoporotic fracture in later life. Genetic variants associated with BMD have been identified by three recent genome-wide association studies. The most significant single-nucleotide polymorphisms (SNPs) from these studies were genotyped to test whether they were associated with peak BMD in premenopausal American women. Femoral neck and lumbar spine BMD were determined by dual-energy X-ray absorptiometry in two groups of premenopausal women: 1524 white women and 512 black women. In premenopausal white women, two SNPs in the *C6orf97*/*ESR1* region were significantly associated with BMD (*p* < 4.8 × 10^−4^), with suggestive evidence for *CTNNBL1* and *LRP5* (*p* < .01). Evidence of association with one of the two SNPs in the *C6orf97*/*ESR1* region also was observed in premenopausal black women. Furthermore, SNPs in *SP7* and a chromosome 4 intergenic region showed suggestive association with BMD in black women. Detailed analyses of additional SNPs in the *C6orf97*/*ESR1* region revealed multiple genomic blocks independently associated with femoral neck and lumbar spine BMD. Findings in the three published genome-wide association studies were replicated in independent samples of premenopausal American women, suggesting that genetic variants in these genes or regions contribute to peak BMD in healthy women in various populations. © 2010 American Society for Bone and Mineral Research.

## Introduction

Osteoporosis is a common skeletal disease characterized by reduced bone mineral density (BMD). After the age of 50 years, the lifetime risk of osteoporotic fractures is approximately 40% in white American women and 14% in white American men.(1) A major predictor of future osteoporotic fracture risk is peak BMD obtained during early adulthood.(2) Although attainment of peak BMD is determined by both genetic and environmental components, twin and family studies indicate that genetic factors account for up to 80% of normal variation in BMD.(3)

Recent genome-wide association studies have identified single-nucleotide polymorphisms (SNPs) associated with BMD.(4–6) Several of the associated genes, such as the *estrogen receptor 1* (*ESR1*), *low-density lipoprotein–related protein 5* (*LRP5*), and *osteoprotegerin* (*OPG*), have well-characterized roles in skeletal biology. However, these studies also have identified novel genes that were not known previously to affect BMD but may play an important role in bone accrual or loss.

The three genome-wide association studies were published at the start of the current study. They were performed on the Framingham family sample of approximately equal proportions of men and women,(4) a UK twin sample of only women,(5) and an Icelandic sample consisting of over 85% women.(6) However, since all three samples had a mean age greater than 50 years, the associations identified may reflect a combination of age-related bone loss and peak BMD. Thus the aim of this study was to replicate the most significant SNPs identified in these three studies in samples of premenopausal women.

## Subjects and Methods

### Sample

Families with two or more healthy premenopausal sisters were recruited from the state of Indiana to identify genes contributing to bone mass. These provided a sample of 1524 American white and 512 American black women (Table 1). A detailed medical history of the siblings was obtained through administration of health and lifestyle questionnaires. Parents of the siblings were recruited to obtain blood for DNA extraction but did not undergo phenotypic assessment. Studies were performed at the Indiana Clinical Research Center. The study was approved by the Institutional Review Board of Indiana University–Purdue University Indianapolis. Informed written consent was obtained from all subjects prior to their participation in the study.

**Table 1:** Sample Characteristics

	White women	Black women
Number of families	762	236
Number of sibling subjects	1524	512
Age, years^a^	33.1 ± 7.2	33.0 ± 6.6
Height, cm^a^	165.4 ± 6.1	164.4 ± 6.2
Weight, kg^a^	70.0 ± 16.6	81.9 ± 19.8
Lumbar spine BMD, g/cm^2^^a^	1.275 ± 0.141	1.339 ± 0.142
Femoral neck BMD, g/cm^2^^a^	1.021 ± 0.136	1.110 ± 0.145

a Mean ± SD.

Sisters were between the ages of 20 and 51 years. Sisters were required to be premenopausal and within 10 years of each other in age. Women who had irregular menses or a history of pregnancy or lactation within 3 months prior to enrollment were excluded. Women taking oral contraceptives were not excluded. Additional exclusion criteria included a history of chronic disease, use of medications known to affect bone mass or metabolism, and an inability to have BMD measured because of obesity.

### BMD, height, and weight measurements

Areal BMD (g/cm^2^) at the lumbar spine (vertebrae L_2_ to L_4_) and femoral neck was measured by dual-energy X-ray absorptiometry (DXA) using two DPX-L and one Prodigy instrument (GE Lunar Corp., Madison, WI, USA). All three instruments were cross-calibrated weekly using a step-wedge phantom. There was no detectable systematic difference between the three machines over the course of the study. The coefficient of variation on duplicate measurements after repositioning the subject was 1.0% for femoral neck and 0.52% for lumbar spine. Siblings were measured on the same instrument usually at the same visit. Height and weight were measured using a Harpenden stadiometer (Holtain Ltd., Crymych, UK) and a Scale-Tronix (White Plains, NY, USA) weighing scale, respectively.

### SNP Genotyping

The most significant SNPs for replication were selected from the three published genome-wide association studies available at the time this study was initiated. The top 25 SNPs for lumbar spine BMD and the top 25 SNPs for femoral neck BMD were selected from the studies of the TwinsUK cohort(5) and an Icelandic sample.(6) Since the genome-wide association study from the Framingham Heart Study reported association results using both men and women,(4) we selected the 20 most significantly associated SNPs each for femoral neck and lumbar spine BMD from a reanalysis of these data in only women (http://www.ncbi.nlm.nih.gov/projects/gap/cgi-bin/study.cgi?study_id = phs000007.v5.p3). This secondary analysis was performed to maximize the likelihood of identifying genes that affect BMD in women. Because 6 of the 140 SNPs were significant in both BMD phenotypes, 134 unique SNPs were tested for replication.

When an SNP failed to work in genotyping assays, another SNP was selected as a replacement using the genotypic information of the CEPH Utah sample in HapMap. The criteria for selection of the replacement SNP was the closest SNP in physical distance to the original SNP (and always within 20 kb), strong linkage disequilibrium (*r*^2^ > 0.5) with the original SNP, and minor allele frequency within 0.1 of the original SNP. When no SNPs met these criteria, then the SNP with the highest *r*^2^ was chosen from all SNPs within 20 kb. The complete list of SNPs tested is provided in Supplemental Table 1.

Genotyping was performed using the iPLEX genotyping assays on the MassARRAY platform, which is based on allele-specific primer extension with mass-modified terminators (Sequenom, Inc., San Diego, CA, USA). The average missing rate for the genotyping assays was 2.1%, with a range from 0.6% to 8.2% (Supplemental Table 1). Using one randomly selected individual in each family, each SNP was tested for significant (*p* < .001) deviation from Hardy-Weinberg equilibrium. In addition, parental genotypes were used to identify Mendelian inconsistencies using the program PedCheck.(7) Inconsistent genotypes were reviewed individually, and the minimum number of genotypes was removed to resolve the Mendelian inconsistencies.

### Genome-wide association data

A genome-wide association study was completed recently in the same sample of 1524 premenopausal white sisters.(8) Thus, for those regions where the previous associations were replicated, the data were augmented with those generated by the genome-wide association study.

Genotyping was performed on the Human610Quadv1_B (Illumina, San Diego, CA, USA) by the Center for Inherited Disease Research (CIDR) using the Illumina Infinium II assay protocol. Genotype calls were made when a genotype yielded a quality metric (Gencall score) of 0.15 or higher. Blind duplicate reproducibility was 99.99% based on 36 paired samples. Samples having genotypes for at least 98% of the SNPs were included in the analyses. Our final analytic sample included 1524 women from 762 different families. This data set demonstrated minimal evidence of stratification, as indicated by a lack of inflation in genome-wide association study *p* values (λ = 1.003) for both the spine and neck BMD phenotypes.

SNPs with a call rate of 98% or greater (*n* = 581,255) were included and subjected to further quality-control analyses. From these, SNPs were removed if either the minor allele frequency was less than 0.01 in this data set (*n* = 32,948) or there was significant deviation (*p* < .00001) from Hardy-Weinberg equilibrium (*n* = 1998). The final data set for analysis consisted of 547,971 SNPs that passed all quality-control measures.

### Statistical analysis

In these samples of women, age and weight were significant covariates of BMD.(9,10) Therefore, regression residuals representing age- and weight-adjusted BMD values for each subject were computed separately in white women and black women and used in all analyses.

A population-based association test was performed using a linear regression framework. SNP genotypes were modeled as taking on three levels (0, 1, and 2) corresponding to the observed genotypes, with BMD as the dependent variable. This model was fitted using the qfam-total analysis in PLINK.(11) Correlation between siblings in the same family was corrected for by obtaining empirical *p* values for the association test using a permutation approach.(10) Permuted replicates were generated by randomly assigning phenotype values among family members, thus preserving the phenotypic correlations and linkage disequilibrium structure from one replicate to another. *p* Values were estimated empirically using 10^5^ permutations.

The significance threshold for the association tests was derived using the simpleM method.(12,13) The effective SNP count for this set of SNPs based on our genotype data was 105, providing a corrected threshold for significant association *p* value of 4.8 × 10^−4^. *p* Values less than .01 were considered suggestive evidence of association. Effect size estimates correcting for sibling correlation were obtained using a mixed-model approach.

For genes or chromosomal regions with SNPs showing replication in white women, the data set was augmented with SNPs from our genome-wide association study to allow more rigorous evaluation of the association. The haplotype block structure was examined with HAPLOVIEW,(14) with blocks defined using the method of Gabriel and colleagues.(15) Linkage disequilibrium confidence interval parameters of (0.5, 0.75) were employed, with recombination and informative fraction values of 0.5 and 0.75, respectively. Rates of recombination were determined as described previously.(16) The blocks then were examined regarding their independent and combined effect on BMD, as measured by the traditional *r*^2^ measure of proportion of phenotypic variance explained.

## Results

### Replication of published genome-wide association studies

White and black women were comparable in age and height (Table 1). However, black women were heavier than white women and had higher BMD values at both the femoral neck and the lumbar spine. In these samples, 129 of the 134 possible SNPs from the three published genome-wide association studies(4–6) had a greater than 90% call rate and were analyzed for association with BMD (Supplemental Table 1). Two SNPs, rs1406493 and rs10496481, were not in Hardy-Weinberg equilibrium (*p* < .001).

Five chromosomal regions associated with BMD in the three genome-wide association studies showed evidence of association (*p* < .01) (Table 2). However, only SNPs in a region harboring *C6orf97* and *ESR1* reached significance level corrected for multiple testing. Only rs851993 in *ESR1* was replicated in both white and black premenopausal women. The study design had 80% power to detect an effect size of 1.4% of total BMD variation in the white sample and 2.3% in the black sample.

**Table 2:** Replication of Significant SNPs Identified in the Previous Genome-wide Association Studies

				White women					Black women				
					
					Femoral neck		Lumbar spine			Femoral neck		Lumbar spine	
									
SNP	Gene^b^	Chr. Position	Chr	MAF^d^	*p* Value	*r*^2^	*p* Value	*r*^2^	MAF^d^	*p* Value	*r*^2^	*p* Value	*r*^2^
rs4870044	*C6orf97*	151,943,102	6	0.30			5.6 × 10^−4^	1.21%					
rs6929137	*C6orf97*	151,978,370	6	0.34	3.8 × 10^−3^	0.25%							
rs7752591	*C6orf97*	151,988,761	6	0.46	**2.9 × 10**^**−4**^	0.11%	2.2 × 10^−3^	0.72%					
rs6900157	*C6orf97*	151,995,820	6	0.36	7.6 × 10^−3^	0.01%	3.2 × 10^−3^	0.86%					
rs851993	*ESR1*	152,047,704	6	0.36	2.6 × 10^−3^	0.40%	**3.3 × 10**^**−4**^	0.40%	0.12	**4.5×10**^**−4**^	0.67%		
rs3020331	*ESR1*	152,050,473	6	0.42			3.7 × 10^−3^	1.24%					
rs851982	*ESR1*	152,066,678	6	0.38			8.9 × 10^−3^	1.29%					
rs4811196	*CTNNBL1*	35,903,108	20	0.19	6.9 × 10^−4^	0.61%							
rs2179320	*CTNNBL1*	35,905,199	20	0.19	3.0 × 10^−3^	0.57%							
rs910760	*CTNNBL1*	35,933,409	20	0.25	1.8 × 10^−3^	0.21%							
rs3736228	*LRP5*	67,957,871	11	0.13			4.5 × 10^−3^	0.40%					
rs10876432 (rs2886129^a^)	*SP7*	52,018,251	12						0.45	6.6 × 10^−3^	3.99%	5.3 × 10^−3^	5.00%
rs6535028	Intergenic	135,757,169	4						0.14	8.0 × 10^−3^	1.18%		

a SNP genotyped as a replacement.

b The nearest genes located within 25 kb.

c Chromosome positions are from NCBI dbSNP Build 130.

d Race-specific minor allele frequencies (MAFs).

e *p* Values and *r*^2^ are shown only for SNPs with *p* < .01. *p* Values that met the corrected significance level of *p* < 4.8 × 10^−4^ are in boldface.

#### White women

The sample of white women replicated association in three different regions. Seven SNPs in a 125-kb region that included neighboring *C6orf97* and *ESR1* were associated with BMD (*p* < .01). However, only two SNPs (rs7752591 and rs851993) met a more stringent level of significance (*p* < 4.8 × 10^−4^). All but rs6929137 in *C6orf97* were associated with lumbar spine BMD, and four SNPs were associated with femoral neck BMD. The estimated effect size on lumbar spine and femoral neck BMD for each SNP individually was no more than 1.3%.

A nonsynonymous *LRP5* SNP (rs3736228) was marginally associated with lumbar spine BMD (*p* = 4.5 × 10^−3^). Three SNPs in *CTNNBL1* also showed suggestive evidence of association with femoral neck BMD (*p* ≤ 3.0 × 10^−3^). These three SNPs, spanning 30 kb, were in high linkage disequilibrium (*r*^2^ > 0.66).

#### Black women

One SNP (rs851993) in the *C6orf97*/*ESR1* region that also replicated in the white women had the most significant association with femoral neck BMD in black women (*p* = 4.5 × 10^−4^). In addition, one SNP in *SP7* (rs2886129) on chromosome 12 showed suggestive evidence of association with both femoral neck (*p* = 6.6 × 10^−3^) and lumbar spine BMD (*p* = 5.3 × 10^−3^). An intergenic SNP (rs6535028) on chromosome 4 was marginally associated with femoral neck BMD (*p* = 8.0 × 10^−3^). None of the SNPs in the *CTNNBL1* or *LRP5* region replicating in the sample of white women demonstrated evidence of association with either BMD phenotype in black women (*p* > .01).

### Supporting evidence from our genome-wide association study

For the three regions associated in the sample of white women, data from our genome-wide association study(8) were reviewed to assess whether there was further support for the association.

#### CTNNBL1

**Fig. 1:**
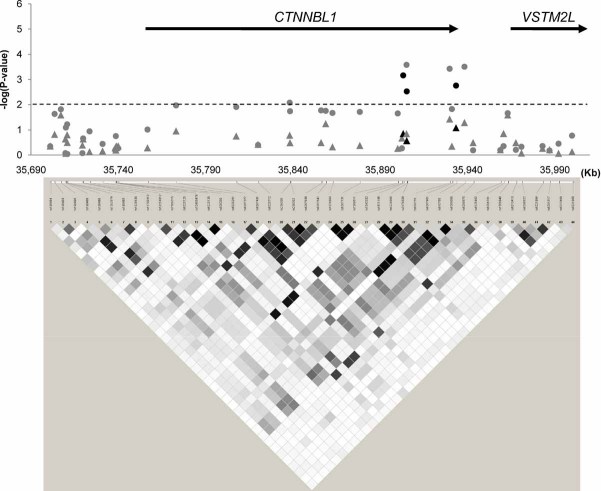
Evidence of association in the *CTNNBL1* region. Arrows indicate the size and location of the genes. Circles (femoral neck) and triangles (lumbar spine) denote SNPs tested in white women in our replication study (*black*) and genome-wide association study (*gray*). Association results (*top panel*) are indicated by chromosomal location on the *x* axis and –log of *p* values on the *y* axis. Threshold of *p* = .01 is marked by the dashed line. The extent of linkage disequilibrium (*r*^2^) structure (*bottom panel*) is represented by the degree of shading. Darker shades indicate greater linkage disequilibrium.

Four additional SNPs from our genome-wide association study supported evidence of the association between femoral neck BMD and *CTNNBL1* (Fig. 1). The region harboring associated SNPs spanned about 100 kb. The most significant SNPs were located in the 3' end of the gene and had minor allele frequency (MAF) of about 0.20 to 0.25.

#### LRP5

**Fig. 2:**
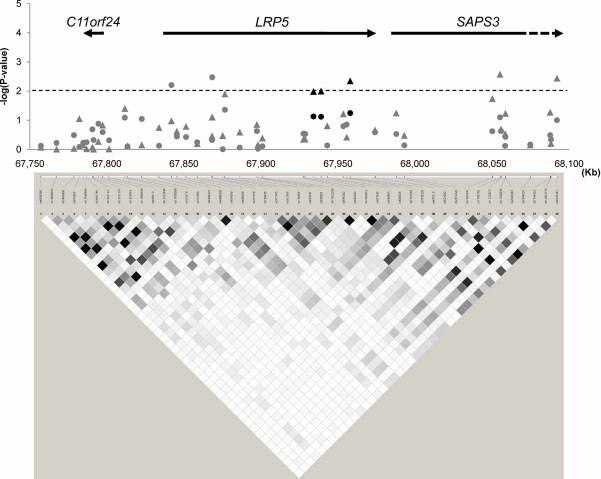
Evidence of association in the *LRP5* region. Arrows indicate the size and location of the genes. Circles (femoral neck) and triangles (lumbar spine) denote SNPs tested in white women in our replication study (*black*) and genome-wide association study (*gray*). Association results (*top panel*) are indicated by chromosomal location on the *x* axis and –log of *p* values on the *y* axis. Threshold of *p* = .01 is marked by the dashed line. The extent of linkage disequilibrium (*r*^2^) structure (*bottom panel*) is represented by the degree of shading. Darker shades indicate greater linkage disequilibrium.

In addition to the association with lumbar spine BMD, there was an association between femoral neck BMD and two SNPs located in the 5' end of the gene (Fig. 2). Two SNPs in the neighboring *SAPS3* gene also were associated with lumbar spine BMD. These SNPs were in moderate linkage disequilibrium with the replicated *LRP5* SNP (rs3736228).

#### C6orf97/ESR1

**Fig. 3:**
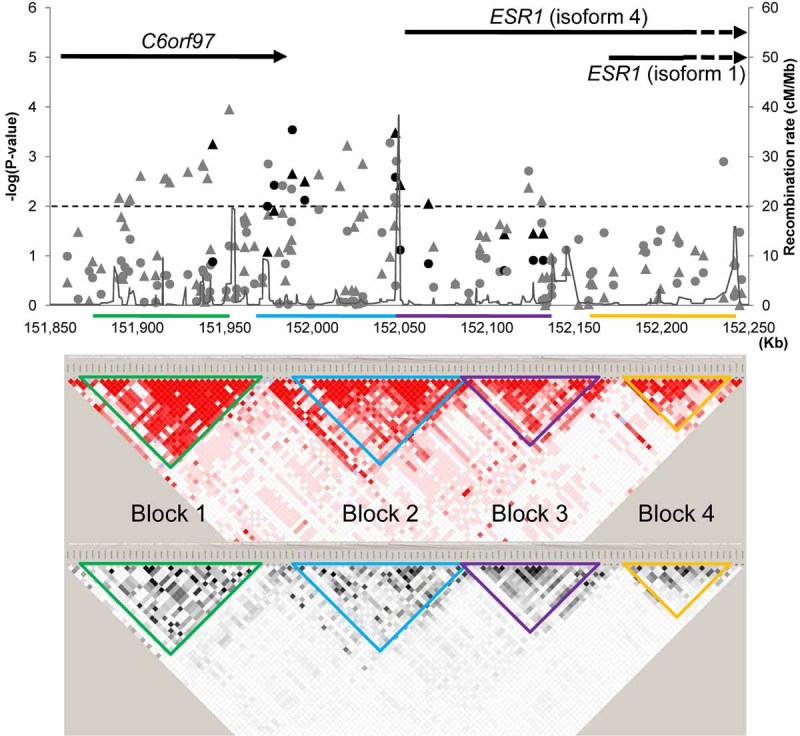
Evidence of association in the *C6orf97*/*ESR1* region. Arrows indicate the size and location of the genes. Circles (femoral neck) and triangles (lumbar spine) denote SNPs tested in white women in our replication study (*black*) and genome-wide association study (*gray*). Association results (*top panel*) are indicated by chromosomal location on the *x* axis and –log of *p* values on the left *y* axis. Threshold of *p* = .01 is marked by the dashed line. Peaks indicate historic recombination in this region. Recombination rates are shown on the right *y* axis. The extent of linkage disequilibrium structures (*middle, D*' and *bottom, r*^2^) is represented by the degree of shading. Darker shading indicates stronger correlation between SNPs. Blocks based on linkage disequilibrium are represented by inverted triangles. The chromosomal regions corresponding to these blocks are marked by colored horizontal lines (*top panel*).

A total of 170 SNPs spanning 622 kb around *C6orf97* and *ESR1* were genotyped in our genome-wide association study. Thirty-three SNPs spanning 346 kb provided evidence of association with BMD (Fig. 3). Closer examination of the association, allele frequency, recombination, and linkage disequilibrium data uncovered a complex pattern of association in this region. Based on the linkage disequilibrium and recombination rate, this region can be divided into four distinct genomic blocks independently associated with femoral neck and/or lumbar spine BMD.

The block most distal to *ESR1* (ie, within *C6orf97*) was defined by SNPs rs9371528 and rs6925996, and 13 SNPs in this block all were associated with lumbar spine BMD. The most significant SNP in this block was rs6925996 (*p* = 1.1 × 10^−4^), which had an MAF of 0.31; it had moderate to high linkage disequilibrium with all the associated SNPs in this block.

The second block, marked by SNPs rs900195 and rs851993, showed a complex pattern of association. Thirteen SNPs were associated with either lumbar spine or femoral neck BMD, and four of those were associated with both phenotypes. SNP rs7752591, located in the center of the block, was the most significant SNP for femoral neck BMD (*p* = 7.0 × 10^−5^), whereas the SNP most proximal to *ESR1* (rs851993) was most significant for lumbar spine BMD (*p* = 2.2 × 10^−4^). Both SNPs also were associated with the other phenotype.

The block between SNPs rs9383939 and rs2504065 (block 3) is adjacent to block 2 and extended from immediately upstream of the long isoform of *ESR1* to its intragenic region. Six SNPs in this block were associated with lumbar spine BMD; two of these SNPs also were associated with femoral neck BMD. All associated SNPs in this block, except one, were relatively common, with MAFs of 0.34 to 0.50. The most significantly associated SNPs with lumbar spine and femoral neck BMD were rs3020331 (*p* = 4.1 × 10^−3^) and rs851991 (*p* = 1.3 × 10^−3^), respectively.

The last block (block 4), spanning SNPs rs17081685 and rs9322336, was located at the beginning of the main *ESR1* isoform. Only one SNP (rs1709183) was associated with femoral neck BMD (*p* = 1.3 × 10^−3^) and had an MAF of 0.29.

**Fig. 4:**
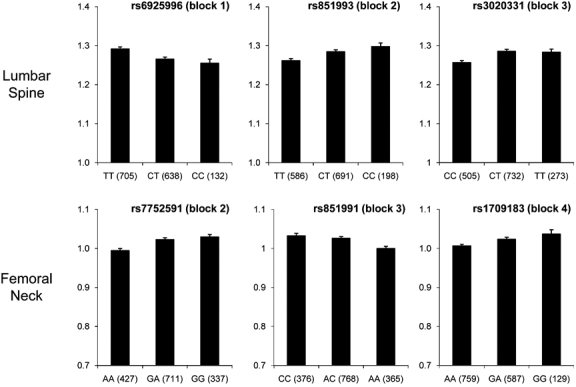
Comparison of mean BMD as a function of SNP genotypes. The most significant SNP for lumbar spine and femoral neck BMD was selected from each of the four different blocks in Fig. 3. Genotypes and corresponding sample numbers (in parentheses) are on the *x* axis; mean BMD ± SEM (g/cm^2^) is on the *y* axis.

The most significant SNPs in each block provided evidence of independent effects on BMD (Fig. 4). The most significant SNPs in each block had little linkage disequilibrium with the most significant SNPs in other blocks (*r*^2^ < 0.4 for spine BMD, *r*^2^ < 0.2 for neck BMD; Table 3). In block 1, the more frequent allele was associated with increased lumbar spine BMD, whereas in blocks 2 and 3, the minor allele was associated with increased BMD. Similarly, in blocks 2 and 4, the minor allele was associated with high femoral neck BMD, whereas in block 3, the major allele was associated with increased BMD. Furthermore, when analyzed jointly, these SNPs explained a significantly greater proportion of BMD variability than each SNP did individually. Each SNP had a relatively small effect size (1.5% or less); however, when analyzed jointly, the simultaneous 3-SNP effect was essentially additive, with the individual SNP summed effect sizes accounting for 71% and 84% of the combined effect on spine and neck BMD, respectively (Table 3). Nonindependent effects would be expected to produce a summed effect much larger than the combined effect. Our results indicate that the effect of each block (and SNPs within) is largely independent of one another.

**Table 3:** Additive Effects of the Most Significant SNPs From Separate Blocks

SNP	Observed effect size (*r*^2^)	Expected effect size (*r*^2^)	Linkage disequilibrium (*D*')	Linkage disequilibrium (*r*^2^)
Lumbar spine				
rs6925996 rs851993 rs3020331	2.18%	3.08%		
rs6925996 rs851993	1.82%	2.14%	0.363	0.035
rs6925996 rs3020331	1.82%	2.07%	0.263	0.024
rs851993 rs3020331	1.43%	1.94%	0.675	0.356
rs6925996	1.13%			
rs851993	1.00%			
rs3020331	0.94%			
Femoral neck				
rs7752591 rs851991 rs1709183	2.52%	3.01%		
rs7752591 rs851991	1.73%	2.28%	0.353	0.108
rs7752591 rs1709183	2.08%	2.07%	0.005	0
rs851991 rs1709183	1.75%	1.68%	0.025	0
rs7752591	1.34%			
rs851991	0.94%			
rs1709183	0.73%			

A more detailed analysis of these blocks uncovered the fact that even the associated SNPs within the same block had only moderate linkage disequilibrium (*r*^2^) owing to significant differences in MAF (data not shown). Even when two physically overlapping groups of SNPs had similar MAFs, they sometimes had little linkage disequilibrium with each other. Thus it is possible that each block has “subblocks” that may affect BMD independently.

## Discussion

In this study we sought to replicate findings from the three published genome-wide association studies(4–6) in our samples of white and black women, who are significantly younger than those in the original studies. Multiple genes/regions were replicated in our sample, indicating that genetic variants in these genes/regions contribute to peak BMD in the general female population. Most of the replicated associations were observed in our white women sample. This is most likely so because the sample of white women was substantially larger than that of black women and therefore had greater power to detect association. In addition, the initial associations were reported in white cohorts consisting primarily of women, and some of the genetic effects may be race-specific. Our sample of black women was smaller than that of white women, resulting in decreased power to detect significant association. However, some of the previously reported associations also were associated in the sample of black women, suggesting that some genes may contribute to variation in BMD across multiple ethnic groups.

Detailed analysis of the *C6orf97*/*ESR1* region demonstrated a complex pattern of association. There were four linkage disequilibrium blocks independently associated with lumbar spine or femoral neck, suggesting the presence of multiple genetic factors affecting peak BMD. Since many of the associated SNPs were located upstream of *ESR1*, these factors likely regulate expression of *ESR1*. This association pattern is in agreement with the previous genome-wide association study in the Icelandic sample, in which the associated SNPs spanning 273 kb were distributed over several linkage disequilibrium blocks, and many of the SNPs had modest to minimal linkage disequilibrium.(6) A similar pattern also was observed in a study of several cancer types, where five haplotype blocks on chromosome 8q24 were associated with the risk of different cancers.(17) These data suggest that high-density SNP genotyping is essential to uncover intricate association patterns because there may be multiple SNPs affecting expression of a gene.

Many of the replicated SNPs are located in or near *ESR1, LRP5*, and *SP7*, genes that have known function in bone. The importance of *ESR1* and *LRP5* in bone biology is clear from Mendelian disorders caused by mutations in these genes,(18–21) meta-analyses of association studies for skeletal phenotypes,(22,23) and phenotypes of genetically modified mice.(24,25) Although our sample of black women is smaller than the white sample (and thus the effect size is likely inflated), the association between BMD and the SNP in *SP7* (*osterix*) supports its candidate gene status as an important regulator of osteoblast differentiation.(26) Of note, the same SNP was marginally associated with femoral neck BMD in white women (*p* = 3.6 × 10^−2^), and *osterix* also was identified in the recent genome-wide association study in a sample of UK children.(27) Thus this study confirms the observed associations with these genes and further strengthens their roles in determination of peak bone mass in young adulthood.

On the other hand, the major advantage of genome-wide association studies is the ability to identify novel genes without a priori knowledge of gene functions. In this regard, *CTNNBL1*, encoding a protein homologous with β-catenin,(28) is one such gene that has no known role in bone biology. Expression of this gene was detected by RT-PCR in all 24 human tissues and cell lines, including bone and osteosarcoma cell lines, MG-63, and SaOS-2 (data not shown). In a yeast two-hybrid system,(29) it also interacts with secreted phosphoprotein 1 (osteopontin), which has a known function in bone remodeling. Although association with the SNPs located in *C6orf97* is likely due to linkage disequilibrium with adjacent *ESR1*, involvement of this open-reading frame in bone cannot be ruled out at present. Similarly, suggestive evidence of association with the intergenic SNP on chromosome 4 should not be overlooked because SNPs like this will provide an opportunity to identify novel genes or regulatory elements including microRNAs. Molecular investigations targeting these genes and regions may open up avenues for new drug targets for osteoporosis.

We have analyzed the white and black women separately because of the documented differences in allele frequency at many of the SNPs considered, along with the BMD differences between the two cohorts. Based on the genome-wide association study,(8) data in the whites are not stratified and are unlikely to produce false-positive associations. To assess the evidence for stratification in our black American sample, we used an admixture covariate derived from principal-component analysis of genotypes at 145 SNPs that we have genotyped previously compared with HapMap controls (YRI and CEU). This analysis demonstrated no appreciable difference in the *p* values for the black American association findings reported and enabled us to rule out stratification as a contributor to the results reported here.

The consistent findings across independent samples of women provide further evidence that the genes or regions replicated are important in determining BMD. The results of this study also showed that certain genes affect both postmenopausal and premenopausal women, suggesting that some genes may act across a wide age range. Alternatively, some of these genes may exert their effects on peak BMD, but the evidence of their effects may persist into older ages. On the other hand, the fact that many of the previously identified regions did not replicate suggests that some of these regions may affect age-related bone loss rather than peak BMD. A further study is necessary to distinguish genetic effects on bone mass and loss.

In summary, we determined that significant SNPs identified in three recent genome-wide association studies also were associated with peak BMD in healthy white and black American women. Replication of the previous genome-wide association studies indicates that variants in *ESR1, LRP5*, and *SP7* affect peak BMD in healthy women and suggests that novel genes such as *CTNNBL1* may have a role in attainment or maintenance of peak BMD. Similar replication studies in more recent genome-wide association studies(27,30,31) are likely to identify additional variants important across multiple ethnicities.
